# Effects of Liver Fibrosis Progression on Tissue Relaxation Times in Different Mouse Models Assessed by Ultrahigh Field Magnetic Resonance Imaging

**DOI:** 10.1155/2017/8720367

**Published:** 2017-01-18

**Authors:** Andreas Müller, Katrin Hochrath, Jonas Stroeder, Kanishka Hittatiya, Günther Schneider, Frank Lammert, Arno Buecker, Peter Fries

**Affiliations:** ^1^Clinic for Diagnostic and Interventional Radiology, Saarland University Medical Center, Kirrberger Str. 100, Bdg. 50.1, 66421 Homburg, Germany; ^2^Department of Medicine, University of California San Diego, 9500 Gilman Dr, La Jolla, CA 92093, USA; ^3^Department of Internal Medicine II, Saarland University, Saarland University Medical Center, Bdg. 77, Kirrberger Str. 100, 66421 Homburg, Germany; ^4^Institute of Pathology, University Hospital Bonn, Bdg. 62, Sigmund-Freud Str. 25, 53127 Bonn, Germany

## Abstract

Recently, clinical studies demonstrated that magnetic resonance relaxometry with determination of relaxation times T1 and T2^⁎^ may aid in staging and management of liver fibrosis in patients suffering from viral hepatitis and steatohepatitis. In the present study we investigated T1 and T2^⁎^ in different models of liver fibrosis to compare alternate pathophysiologies in their effects on relaxation times and to further develop noninvasive quantification methods of liver fibrosis. MRI was performed with a fast spin echo sequence for measurement of T1 and a multigradient echo sequence for determination of T2^⁎^. Toxic liver fibrosis was induced by injections of carbon tetrachloride (1.4 mL CCl_4_ per kg bodyweight and week, for 3 or 6 weeks) in BALB/cJ mice. Chronic sclerosing cholangitis was mimicked using the ATP-binding cassette transporter B4 knockout* (Abcb4* ^−/−^) mouse model. Untreated BALB/cJ mice served as controls. To assess hepatic fibrosis, we ascertained collagen contents and fibrosis scores after Sirius red staining. T1 and T2^⁎^ correlate differently to disease severity and etiology of liver fibrosis. T2^⁎^ shows significant decrease correlating with fibrosis in CCl_4_ treated animals, while demonstrating significant increase with disease severity in* Abcb4* ^−/−^ mice. Measurements of T1 and T2^⁎^ may therefore facilitate discrimination between different stages and causes of liver fibrosis.

## 1. Introduction

Fibrosis, cirrhosis, and ultimately failure are the common sequelae of chronic liver injury [1, 2]. In the past decade detailed insights into the molecular pathology of liver inflammation have been gained [3–5]. Nowadays, efficient therapies are available for chronic damage of hepatocytes caused by persistent viral infection [6]. Yet, the development of pharmaceuticals and new treatment strategies for other causes of the disease is still hampered by the lack of efficient, noninvasive methods for the identification and quantification of liver fibrosis both in humans [6] and in laboratory animals. Ultrasound [7], magnetic resonance techniques [8, 9], and blood tests [10, 11] are available for noninvasive or minimally invasive diagnosis of liver damage. Nonetheless, detection and staging of mild to moderate fibrosis or regression of fibrosis are still difficult to accomplish [9, 12]. In clinical research projects, magnetic resonance relaxometry (MRR) of T1 and T2^*∗*^ has recently been evaluated for staging of liver fibrosis [13] and for the development of a decision tree allowing assessment of early liver disease [14].

In patients with liver fibrosis, iron deposition to the liver might lead to reduction of T1 and subsequent underestimation of disease severity [13, 14]. By correcting for effects of iron deposition, the reliability of fibrosis assessment by T1 mapping can be enhanced and could prove sufficient for routine clinical evaluations [13]. In these and other clinical MRI studies of liver fibrosis, primary biliary cirrhosis (PBC) or primary sclerosing cholangitis (PSC) is usually underrepresented. Both disease entities present with distinct features, when histopathology is compared to liver fibrosis caused by alcohol or viral hepatitis. Consequently, they can be expected to show different results in MRR. Whereas the most common model of PBC based on bile duct ligation has been investigated by MRR [15] in experimental studies, MRR evaluations of animal models of PSC have not been published to date.

In this study we compared one of the standard models of liver fibrosis based on CCl_4_ intoxication with ATP-binding cassette transporter B4 knockout* (Abcb4* ^−/−^) mice, which develop sclerosing cholangitis and biliary fibrosis very similar to PSC in humans, in regard to tissue relaxation times assessed by MRR methods.

## 2. Materials and Methods

Animal experiments were performed with approval by the local authorities, in line with the laws for the protection of animals and by following all institutional and national guidelines for the care and use of laboratory animals. All inbred mouse lines (BALB/cJ) were obtained from Charles River Laboratories (Sulzfeld, Germany).

All mice were housed in individually ventilated cages with a 12 h light-dark cycle; temperature and humidity were regulated to 22 ± 1°C and 55 ± 5%, respectively, with water and standard diet (Altromin 1314, Altromin, Lage, Germany) provided ad libitum.

### 2.1. Animal Models

In this study, liver fibrosis caused by CCl_4_ intoxication and deficiency of the ATP-binding cassette transporter B4 were investigated in their effects on relaxation times T1 and T2^*∗*^.

#### 2.1.1. Sample Size

The sample size needed was estimated by interpretation of results from former MRR studies [15, 16] and calculated with SigmaPlot software (v.13.0, Systat Software GmbH, Erkrath, Germany). The mean T1 value for healthy liver tissue was estimated to be around 1000 ms, with a minimum detectable difference between the treatment groups of 20% (=200 ms) and a standard deviation within the single groups of 12.5% (=125 ms). With a desired power of 0.80 and alpha of 0.05, minimum group size was calculated to include 10 animals.

#### 2.1.2. Model of Liver Fibrosis due to Chronic Intoxication

A total of 22 BALB/cJ inbred mice received repeated intraperitoneal injections of a mixture of CCl_4_ and mineral oil at a dose of 0.7 mL CCl_4_ kg^−1^ of body weight twice a week, beginning at an age of 6 weeks.

Animals were divided into two groups and disease severity was modified by varying the number of CCl_4_ injections. One group (*n* = 11) received six injections in three weeks (referred to as BALB/cJ + 6x CCl_4_), whereas the other group (*n* = 11) received a total number of 12 injections in six weeks (referred to as BALB/cJ + 12x CCl_4_).

MRR experiments were performed 48 h after the final CCl_4_ injection, to minimize the effects of acute inflammation. Untreated age matched BALB/cJ inbred mice (*n* = 11) served as controls.

#### 2.1.3. Model of Liver Fibrosis Based on Genetic Alteration

Since bearing a distinct genetic feature similar to a subset of patients with primary sclerosing cholangitis [17], BALB/cJ mice with the* Abcb4* ^tm1Bor^ knockout [18] were examined and compared to CCl_4_ treated animals. BALB/cJ-*Abcb4* ^−/−^ mice develop moderate to severe fibrosis at 15 to 17 weeks of age.

Genotypes of mice included in the MRR experiments were confirmed by polymerase chain reaction (PCR) of tail DNA using* neo* (5′-CTTGGGTGGAGAGGCTATTC-3′ and 5′-AGGTGAGATGACAGGAGATC-3′) and* Abcb4* (5′-CACTTGGACCTGAGGCTGTG-3′ and 5′-TCAGGACTCCGCTATAACGG-3′) specific primer pairs. The PCR contained 10x PCR buffer (Applied Biosystems, Foster City, CA, USA), 2 mM MgCl_2_, 10 *μ*M dNTPs, 10 *μ*M primer, 1.25 U TaqDNA polymerase (Invitrogen, Darmstadt, Germany), and 20–100 ng of DNA in 25 *μ*L reactions. PCR cycling conditions were 94°C/30 s, 55°C/60 s, and 72°C/30 s for 35 cycles, with a final extension step of 10 min at 72°C.

Eleven BALB/cJ-*Abcb4* ^−/−^ animals were investigated between 15 and 17 weeks of age, while untreated age matched BALB/cJ represented controls.

### 2.2. Magnetic Resonance Experiments

All animals were examined in a horizontal-bore 9.4 T MRI animal scanner (Biospec Avance III 94/20, Bruker Biospin Inc., Billerica, MA, USA) with a BGA12S gradient system (maximum strength 675 mT m^−1^, linear inductive rise time 130 *μ*s, and maximum slew rate 4673 mT/m/s) run with the software package ParaVision 5.1 supplied with the scanner.

#### 2.2.1. Animal Handling

All MRI experiments were performed with the animals under general anesthesia using a mixture of isoflurane and oxygen. Anesthesia was initiated in an induction chamber using a mixture of 3% isoflurane and 97% oxygen. During imaging, anesthesia was maintained with an animal nose mask supplying a mixture of 0.8% to 2% isoflurane and 99.2% to 98% oxygen at a flow rate of 1.5 l min^−1^. All animals were positioned prone in a dedicated animal cradle heated via a thermostat driven water bath. For measuring the core temperature of the animals, a temperature sensor (SA Instruments Inc., Stony Brook, NY, USA) was inserted into the rectum. A pressure transducer consisting of a small air cushion (Graseby infant respiration sensor, Smiths Medical, Dublin, OH, USA) was attached to the abdominal wall to monitor the respiratory rate during the MR procedures. Physiological data were processed and monitored using an external computer with dedicated software (PC-SAM32, SA Instruments, Inc., Stony Brook, NY, USA). The temperature of the thermostat water bath and the amount of isoflurane administered were adjusted manually to facilitate comparable physiological conditions between the different animals, that is, core temperatures of 38.0 +/− 1°C and respiration rates of 30–50 min^−1^ during the imaging experiments.

MRI was performed using a linear polarized coil developed for imaging of the mouse abdomen, with an inner diameter of 38 mm (Bruker Biospin Inc., Billerica, MA, USA).

#### 2.2.2. Magnetic Resonance Relaxometry

**Figure 1:**
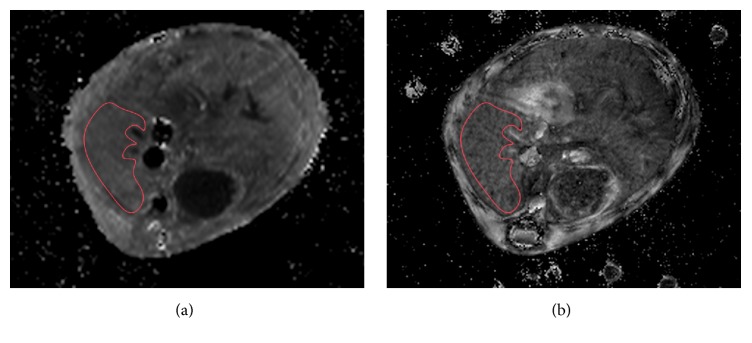
Typical T1 and T2^*∗*^ maps acquired from an animal treated with 12 CCl_4_ injections. Red circles indicate area within the right liver lobe selected for calculating mean T1 and T2^*∗*^ values. (a) T1 map, (b) T2^*∗*^ map.

Relaxometry was performed acquiring single slice datasets in axial orientation, covering the right liver lobe (Figure 1). Datasets for calculating relaxation time T1 of liver tissue were acquired using a RARE approach (rapid acquisition with relaxation enhancement) with repetition times TR = 400 ms, 800 ms, 1200 ms, 1600 ms, and 3000 ms and TE = 7.5 ms (RARE-VTR, Paravision 5.1, Bruker Biospin Inc., Billerica, MA, USA), with a slice thickness of 0.7 mm, a 128 × 128 matrix, a field of view of 25.6 × 25.6 mm^2^, zero fill in phase direction 1.5,   5 averages, and acquisition time 27 min 45 s.

Datasets for determination of relaxation time T2^*∗*^ were generated with a MGE (multigradient echo, Paravision 5.1, Bruker Biospin Inc., Billerica, MA, USA) sequence, with TR set to 1000 ms and multiple TE of 3.5 ms, 7.5 ms, 11.5 ms, 15.5 ms, 19.5 ms, 23.5 ms, 27.5 ms, 31.5 ms, 35.5 ms, 39.5 ms, 43.5 ms, and 47.5 ms, with a slice thickness of 0.7 mm, a 256 × 256 matrix, field of view of 25.6 × 25.6 mm^2^, zero fill in phase direction 1.34,  3 averages, and acquisition time 9 min 36 s.

Following MRR experiments, the animals were sacrificed and liver specimens for histologic and biochemical investigations were collected immediately.

### 2.3. Data Analysis

#### 2.3.1. Determination of Relaxation Time Constants T1 and T2 ^*∗*^

For calculating relaxation times T1 and T2, software modules within the MRI scanner operating software were used (Paravision 5.1, Image Sequence Analysis tool). T1 maps were calculated on a voxel-by-voxel basis from datasets acquired at TR = 400 ms, 800 ms, 1200 ms, 1600 ms, and 3000 ms and TE = 7.5 ms. The relaxation time constant T1 was determined with monoexponential recovery fitting on the individual voxel signal intensity values, using an algorithm based on the Levenberg-Marquardt algorithm for solution of nonlinear least square equations [19], with

(1) $$\begin{array}{c} y=A+C\times \left(\;1-e^{-TR/T1}\;\right) \end{array}$$

with *y* representing the individual pixel SI and *A* and *C* as variables for fitting. Data fitting was performed with absolute bias, allowing variation of *A* and *C* for minimizing the standard deviation of the fit.

T2^*∗*^ maps were generated by performing voxel-by-voxel monoexponential recovery fitting and calculation of the relaxation time constant T2^*∗*^ from datasets acquired at TR = 1000 ms, TE = 3.5 ms, 7.5 ms, 11.5 ms, 15.5 ms, 19.5 ms, 23.5 ms, 27.5 ms, 31.5 ms, 35.5 ms 39.5 ms, 43.5 ms, and 47.5 ms, employing

(2) $$\begin{array}{c} y=A+C\times e^{-TE/T2^{*}} \end{array}$$

with *y* representing SI for the individual pixels within the individual voxel signal intensity values extracted from datasets acquired with the different TE and *A* and *C* again serving as variables for data fitting with absolute bias.

T1 and T2^*∗*^ maps were transferred to a workstation with OsiriX v.4.1.2. 32-bit (Pixmeo Sarl, Geneva, Switzerland), randomized, and anonymized, and the date of imaging was removed. Images were analyzed by a radiologist blinded to animal identity, age, treatment, and genetic status. Regions of interest (ROI) were created in the transferred DICOM files as individual polygons, maximizing the sampled liver tissue while carefully avoiding macroscopic structures such as large blood vessels or bile ducts (Figures 1(a) and 1(b)). T1 and T2^*∗*^ values were calculated as mean from the individual voxels included in the ROI. To minimize sampling error, ROI were placed in the right liver lobe.

#### 2.3.2. Calculating T1/T2 ^*∗*^

T1 measurements are affected by tissue iron content [13], and iron deposition can be quantified indirectly in vivo via T2^*∗*^ measurements [20]. For compensation of iron deposition effects on T1, the T1/T2^*∗*^ quotients were calculated and compared between the different animal groups as well as with results of biochemical and histologic investigations.

### 2.4. Biochemical Characterization of Fibrosis

Hepatic collagen contents were quantified in liver hydrolysates from snap frozen specimens of liver tissue, by colorimetric measurement of the collagen-specific amino acid hydroxyproline [21]. Tissue samples were taken from the right liver lobe. Analyses were performed in duplicates, and for each animal the mean hydroxyproline content was calculated in *μ*g per g fresh liver tissue.

### 2.5. Histopathological Characterization

Liver samples for histopathological evaluation were fixed in 4% neutral buffered formalin at 4°C for 24 h and embedded in paraffin, and tissue sections were generated at a thickness of 5 *μ*m. Sections were examined by light microscopy (DM4000B, Leica Microsystems, Wetzlar, Germany). Histomorphometric analyses were conducted through a high-resolution digital camera attached (DFC 420 C, Leica Microsystems, Wetzlar, Germany), with the use of Leica Application Suite V.4.5.0. software (Leica Microsystems, Wetzlar, Germany).

#### 2.5.1. Assessment of Liver Fibrosis

For assessment of liver fibrosis severity by histology, collagen fibers in paraffin sections were stained with Sirius red [22]. For 40 out of 44 animals, liver fibrosis staging was assessed by a pathologist blinded to the study protocol. Semiquantitative scoring was performed with a system adapted from Batts and Ludwig [23] as well as Ishak et al. [24], principally differentiating the stages (*F*-scores)* F*0 to* F*4 with specific criteria for both animal models. Staging criteria are summarized in Tables 1 and 2.

#### 2.5.2. Quantification of Iron Deposition

Prussian blue (PB) staining was performed for all animals investigated, by incubation of slides in 10% potassium hexacyanoferrate for 10 minutes at room temperature, followed by treatment with 5% potassium hexacyanoferrate in 10% hydrochloric acid for 30 minutes at 37°C, and subsequent rinsing in distilled water. Counterstaining was performed with neutral red for 3 minutes. PB stained liver sections were photographed at ten microscopic fields spanning 400 *μ*m × 500 *μ*m, the number of PB positive cells was counted, and an average was calculated.

### 2.6. Statistical Analysis

Single animal hydroxyproline, T1, T2^*∗*^, and T1/T2^*∗*^ values,* F*-scores, and the relative number of iron positive cells were used to calculate mean values ± SD. Kolmogorov-Smirnov Test was applied on all data for evaluating normal distribution. All values of the different animal subgroups were compared with each other by one-way analysis of variance (ANOVA) with Tukey's multiple comparison test. Pearson correlation coefficients (*r*_*P*_) and corresponding *R*^2^ and *P* values were calculated for* F*-scores and the relative number of iron positive cells as well as for individual T1, T2^*∗*^, and T1/T2^*∗*^ ratio and for hydroxyproline levels. For all tests, values of *P* < 0.05 were regarded as significant. Statistical analyses were performed using GraphPad Prism software (version 6.0, GraphPad Software, San Diego, CA, USA).

## 3. Results

**Figure 2:**
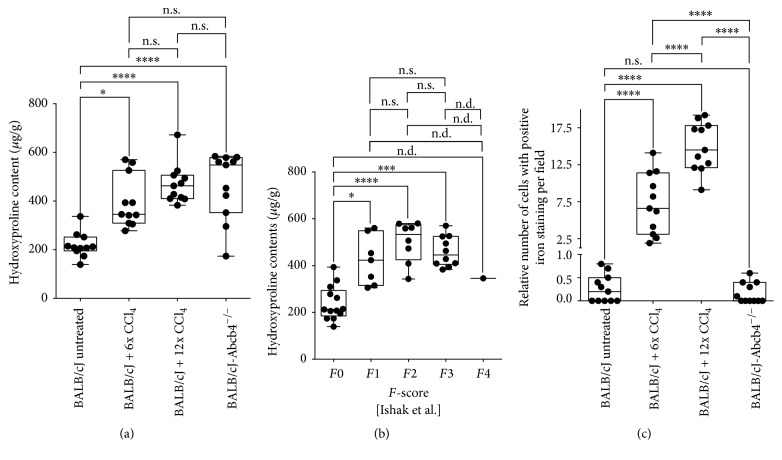
Biochemical and histopathological evaluation of liver fibrosis. Data graphed as median, minimum to maximum with all values, and 25% and 75% percentile label displayed. One-way ANOVA: asterisks indicate  ^*∗*^*P* < 0.01,  ^*∗∗∗*^*P* < 0.001, and  ^*∗∗∗∗*^*P* < 0.0001; n.s.: not significant; n.d.: not determinable. (a) Hepatic hydroxyproline contents stratified according to animal group. (b) Hepatic hydroxyproline contents stratified according to fibrosis score categories* F*0–*F*4. (c) Iron positive cells per field stratified according to animal group.

Hydroxyproline content of liver tissue (Figure 2(a)) varied within the four investigated animal groups. Mean values were highest in animals treated with 12 CCl_4_ injections (470.7 ± 80.9 *μ*g g^−1^) and mice with the* Abcb4* ^−/−^ genotype (437.0 ± 152.1 *μ*g g^−1^). Animals injected six times with CCl_4_ showed an intermediate mean content (396.8 ± 105.7 *μ*g g^−1^), while the lowest average hydroxyproline content was detected for untreated mice (218.8 ± 51.31 *μ*g g^−1^). For all disease models, hydroxyproline levels were significantly different from control animals, with *P* < 0.01 for untreated animals and BALB/cJ + 6x CCl_4_, and *P* < 0.0001, when untreated animals were compared to BALB/cJ + 12x CCl_4_ or BALB/cJ-*Abcb4* ^−/−^ mice. Differences within the treatment groups were not significant for hydroxyproline content.

Severity of liver fibrosis could be assessed by histology for a subset of 40 animals. The histopathological* F*-score showed a moderate correlation with hepatic hydroxyproline content (*r* = 0.55, *R*^2^ = 0.30, and *P* < 0.0001). As shown in Figure 2(b) hepatic hydroxyproline content for* F*0 (nonfibrosis) was significantly lower compared to all other* F*-scores (*F*1: *P* < 0.0001,* F*2: *P* < 0.0001, and* F*3: *P* < 0.0001), excepting* F*4 (*N* = 1), whereas hydroxyproline content within the* F*-score groups (*F*1–*F*3) did not differ significantly.

Histologic analysis of iron deposition (Figure 2(c)) showed significantly higher levels in liver specimen of BALB/cJ + 6x CCl_4_ (7.22 ± 4.10 iron positive cells per field) and BALB/cJ + 12x CCl_4_ (14.94 ± 3.31 iron positive cells per field) treatment groups, when compared to untreated controls (0.26 ± 0.30 iron positive cells per field) and BALB/cJ-*Abcb4* ^−/−^ mice (*P* < 0.0001). In contrast to CCl_4_ treated animals, cells with positive Prussian blue staining are scarce in BALB/cJ-*Abcb4* ^−/−^ tissue samples (0.16 ± 0.22 iron positive cells per field). Differences between both CCl_4_ treatment groups also proved significant (*P* < 0.0001).

**Figure 3:**
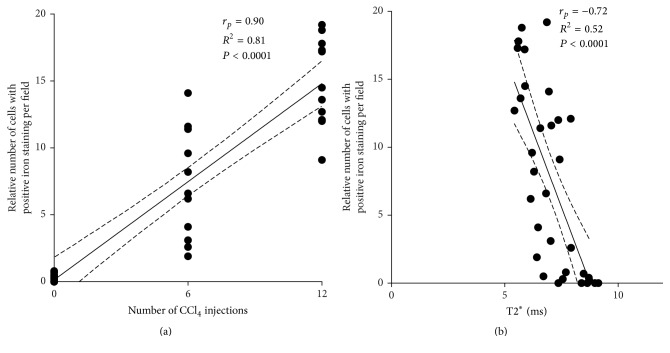
Iron positive cells in liver tissue, CCl_4_ administration, and T2^*∗*^. (a) Plot of the relative number of iron positive cells per field versus number of CCl_4_ doses. (b) Plot of T2^*∗*^ versus number of iron positive cells per field in CCl_4_ treated animals.

This CCl_4_ dose dependent effect is highlighted by a strong positive correlation between the total number of CCl_4_ injections and the relative number of iron positive cells (Figure 3(a), *r* = −0.90, *R*^2^ = 0.81, and *P* < 0.0001). Calculation of Pearson correlation coefficient for the relative number of iron positive cells and individual T2^*∗*^ values demonstrated a concomitant negative correlation (Figure 3(b), *r* = −0.72, *R*^2^ = 0.52, and *P* < 0.0001).

**Figure 4:**
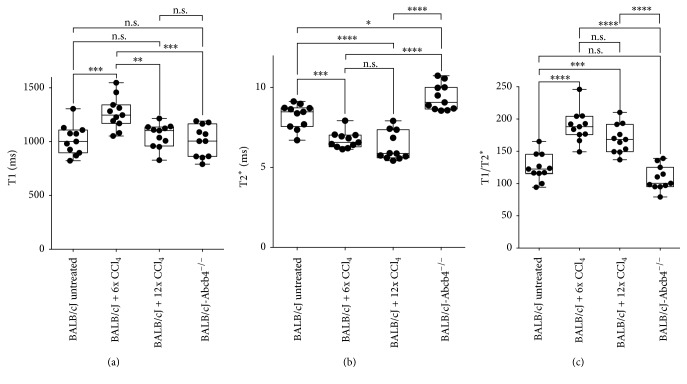
Relaxation times T1 (a), T2^*∗*^ (b), and T1/T2^*∗*^ ratio (c) for the different animal groups. Data graphed as median, minimum to maximum with all values, and 25% and 75% percentile label displayed. Asterisks indicate significant differences demonstrated by one-way ANOVA,  ^*∗*^*P* < 0.01,  ^*∗∗*^*P* < 0.005,  ^*∗∗∗*^*P* < 0.001, and  ^*∗∗∗∗*^*P* < 0.0001; n.s.: not significant.

Mean T1 values (Figure 4(a)) were recorded at 1017 ± 139.2 ms for untreated animals, 1265 ± 148.5 ms for BALB/cJ + 6x CCl_4_, 1056 ± 111.1 ms for BALB/cJ + 12x CCl_4_, and 1014 ± 145.8 ms for BALB/cJ-*Abcb4* ^−/−^ mice.

When mean T1 values for the different experimental groups were compared, a significant difference (*P* < 0.001) was observed between untreated and BALB/cJ mice after six injections of CCl_4_. Compared to animals receiving six injections, mean T1 values of animals treated with 12 CCl_4_ injections and of BALB/cJ-*Abcb4* ^−/−^ mice were significantly lower (*P* < 0.005 and *P* < 0.001).

Mean T2^*∗*^ values (Figure 4(b)) were recorded at 8.217 ± 0.7687 ms for untreated animals, 6.715 ± 0.5179 ms for BALB/cJ + 6x CCl_4_, 6.306 ± 0.8995 ms for BALB/cJ + 12x CCl_4_, and 9.369 ± 0.7647 ms for BALB/cJ-*Abcb4* ^−/−^ mutants. Mean T2^*∗*^ values for BALB/cJ + 6x CCl_4_ and BALB/cJ + 12x CCl_4_ were significantly lower than for untreated mice (*P* < 0.001 and *P* < 0.0001), while BALB/cJ-*Abcb4* ^−/−^ mice show a significantly higher mean T2^*∗*^ value than control animals (*P* < 0.01).

Mean T1/T2^*∗*^ ratios (Figure 4(c)) were calculated at 124.8 ± 20.65 for untreated animals, 189.1 ± 24.87 for BALB/cJ mice injected six times with CCl_4_, 169.3 ± 22.25 for animals that received 12 CCl_4_ treatments, and 108.9 ± 17.50 for BALB/cJ-*Abcb4* ^−/−^ animals. Results for both mice treated six and 12 times with CCl_4_ proved to be significantly different from mean T1/T2^*∗*^ ratio determined for untreated animals (*P* < 0.001 and *P* < 0.0001). Also, when compared to T1/T2^*∗*^ ratios calculated for BALB/cJ-*Abcb4* ^−/−^ animals (*P* < 0.0001 for both groups), T1/T2^*∗*^ ratio for mice treated six and 12 times with CCl_4_ was significantly higher.

**Figure 5:**
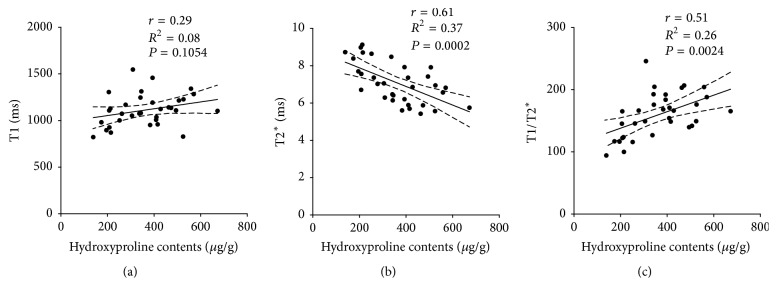
Plot of relaxation times T1 (a), T2^*∗*^ (b), and T1/T2^*∗*^ ratio (c) versus hepatic hydroxyproline contents for CCl_4_ treated animals and controls.

Correlations between MR relaxometry and tissue characterization results were highest for hydroxyproline contents. Calculation of Pearson correlation coefficient for relaxation times and hydroxyproline levels for CCl_4_ treated animals and controls (Figure 5) demonstrated moderate correlations for T2^*∗*^ and hydroxyproline content (Figure 5(b), *r* = −0.61, *R*^2^ = 0.37, and *P* = 0.0002) as well as for T1/T2^*∗*^ and hydroxyproline content (Figure 5(c), *r* = 0.51, *R*^2^ = 0.26, and *P* = 0.0024).

**Figure 6:**
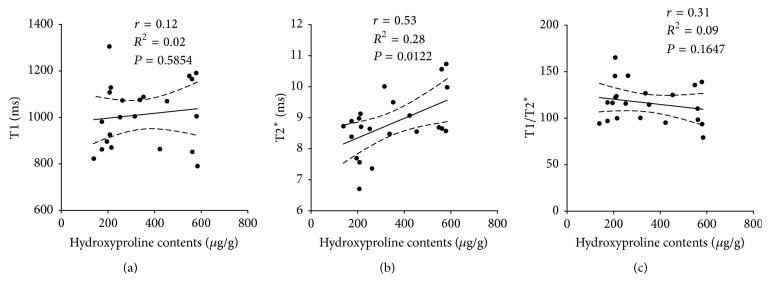
Plot of relaxation times T1 (a), T2^*∗*^ (b), and T1/T2^*∗*^ ratio (c) versus hepatic hydroxyproline contents for BALB/cJ-*Abcb4* ^−/−^ mice and control animals.

From determination of Pearson correlation coefficients for relaxation times and hydroxyproline content for BALB/cJ-*Abcb4* ^−/−^ mice and untreated controls (Figure 6), a moderate correlation was found for T2^*∗*^ and hydroxyproline content (Figure 6(b), *r* = 0.53, *R*^2^ = 0.28, and *P* = 0.0122).

## 4. Discussion

The aim of our study was to compare by parametric magnetic resonance imaging the standard model of liver fibrosis based on CCl_4_ intoxication and the ATP-binding cassette transporter B4 knockout* (Abcb4* ^−/−^) mouse model of PSC and to relate results to the severity of liver fibrosis.

As standard for determining the severity of liver fibrosis, we favored tissue collagen content over histological investigations, since histological fibrosis staging is a less quantitative measuring method for liver fibrosis [25–27]. This assumption is supported by our results showing that the quantitative biochemical criterion of liver tissue hydroxyproline content does correlate significantly with results of semiquantitative histopathology. In agreement with our theory, liver collagen contents were significantly higher in all ABCB4 deficient and CCl_4_ treated mice, when compared to control animals (Figure 2(a)). Analyzing and comparing hepatic collagen contents between animals after six and 12 injections of CCl_4_ showed the expected increase, thereby confirming tissue hydroxyproline as a quantitative marker for liver fibrosis. However, while a moderate correlation between fibrosis stages (*F*-scores) and hydroxyproline levels can be found, both median (Figure 2(b)) and mean liver hydroxyproline values for animals grouped according to their fibrosis scores (Figure 2(b)) do not differ significantly between fibrosis stages* F*1,* F*2, and* F*3. Also, significance could not be demonstrated for the differences in hydroxyproline content between the different treatment groups with suspected liver damage (Figure 2(a)).

These observations do reflect the intrinsic problems of fibrosis staging by biochemical and histopathological methods, in particular the scattering of measured data due to sampling error and individual variation in laboratory animals. Such effects have been well experienced (for the CCl_4_ intoxication model of liver fibrosis) by other investigators [28]. The resulting problem can be resolved either by subsuming different disease severity groups, that is, comparison of disease stages* F*0,* F*1 +* F*2, and* F*3 +* F*4 [28] or by increasing the sample size [29]. Unfortunately, the vast majority of animals in our investigation were staged at* F*1 to* F*2, and animal experiments could not be expanded due to limited resources and permissions. Yet, histologic investigation of iron deposition clearly demonstrates the dose dependent tissue damage caused by CCl_4_ administration (Figure 3(a)). Problems associated with conventional biochemical and histologic methods for fibrosis staging stress the need for more robust, yet sensitive and preferably noninvasive fibrosis staging methods for preclinical research.

Initially it has been assumed that the changes of tissue morphology and physiology associated with chronic inflammation and scarring of the liver lead to an increase of T1 [30–32] and could be suitable for determining the various stages of liver disease. Most studies on liver relaxation times in diseased animals and humans have demonstrated some kind of increase in T1 [12–15, 33, 34], most often interpreted as a result of liver edema due to more or less persistent chronic inflammation. Contrary to these findings, other investigators have not been able to show stable or progressive increases in T1 [35, 36]. In a broad set of patients with liver fibrosis investigated at a magnetic field strength of 3 T, Banerjee et al. [13] have observed a strong effect of tissue iron content on T1 measurements, while calculation of an effective (corrected) T1 based on Bloch simulations in the presence of excess tissue iron resulted in stronger correlation with fibrosis score than with unprocessed T1 relaxation times. It was concluded by Banerjee et al. that T1 mapping with subsequent correction for effects of deposited iron may turn MRR into a practical method for fibrosis assessment.

The lack of relevant numbers of patients with PBC or PSC as the primary cause of fibrosis in this study has to be mentioned. These disease forms present with distinct features, when histopathology is compared with more common causes of liver fibrosis such as alcoholic liver disease or chronic viral hepatitis.

Whereas the most common model of PBC, bile duct ligation in the rat, has been investigated by T1 MRR in an experimental study at 4.7 T [15], MRR evaluations of animal models similar to PSC in humans to date have not been published. Here we present, to our knowledge, the first MRR study on animals with ABCB4 transporter deficiency.

For the standard intoxication model used in this study as a control, we observed an increase of T1 levels > 25% in animals treated with six injections of CCl_4_, while in animals treated with 12 injections, T1 relaxation time remains effectively unchanged. We attribute this effect (analogous to the observations by Banerjee et al. [13]) to iron deposition in diseased liver tissue, since the relative number of cells with positive Prussian blue staining correlates positively with the number of CCl_4_ injections (Figure 2(c)). However, decline of T1 with severity of fibrosis seems to be much more pronounced in our experimental settings than in the clinical investigations performed recently [13, 14]. We interpret this decline mainly as a result of the higher static magnetic field strength of the MR scanner employed in our study. Since the actual algorithm for compensation of iron effects is patent protected (Tunnicliffe E, inventor, UK Provisional Patent 1304728.7, March 15, 2013), we were not able to apply the exact correction procedure as employed by Banerjee et al. [13] for T1 measurements. However, relating T1 measurements to tissue iron content by introducing the T1/T2^*∗*^ ratio as new parameter resulted in stronger correlation with tissue hydroxyproline levels in CCl_4_ treated animals and controls when compared to initial T1 measurements itself.

The number of positive Prussian blue staining cells was not increased in* Abcb4* ^−/−^ mice (Figure 2(c)). In humans, loss of function of the ABCB4 transporter can lead to biliary cirrhosis resembling progressive PSC [17]. Mice bearing a homozygous defective genotype present with distinct pathophysiology of progressive liver fibrosis when compared to intoxication models like CCl_4_ treatment [37]. Hence, MRR results from BALB/cJ-*Abcb4* ^−/−^ mice differ profoundly from those from CCl_4_ treated animals. While statistical analysis shows no significant difference between mean T1 values for controls and knockout animals, T2^*∗*^ values are significantly higher for ABCB4 deficient animals than for control mice. The fact that T2^*∗*^ is not decreasing can be explained by the low amount of iron deposits. However, there is no explanation why T2^*∗*^ is increased in this model. Correlating hydroxyproline levels with different MRR parameters (T1, T2^*∗*^, and T1/T2^*∗*^) for a group consisting of untreated controls and BALB/cJ-*Abcb4* ^−/−^ mice yielded a significant positive correlation for T2^*∗*^ and T1/T2^*∗*^ but not for T1. From our experiments, the (biochemical) cause for unchanged mean T1 values in ABCB4 deficient mice cannot be deduced. Theoretically, an increase of T1 values caused by edema would be expected. Hypothetically, an increase in fat or bile acid content could be responsible for counteracting the increase in T1, thereby simulating unchanged T1 values. The same changes (increase in lipid contents) could be responsible for the observed increase in T2^*∗*^ values. In summary, changes of MR parameters depend not only on the existence of liver fibrosis but also on the etiology of the disease. Therefore, mathematical correction algorithms introduced for the improved correlation of MR parameters should only be accepted for the corresponding etiology and not for liver fibrosis in general. Furthermore, basing decisions on clinical interventions like liver biopsies on individual T1 or T2^*∗*^ measurements might lead to overlooking of liver fibrosis forms caused by impairment of the hepatobiliary system, especially at early stages.

In our study, higher mean T1/T2^*∗*^ ratios could identify liver fibrosis caused by chronic intoxication. A decreased mean T1/T2^*∗*^ ratio was a sign for ABCB4 deficiency. While this observation warrants further investigation of MRR for differential diagnosis of liver disease, it is noteworthy that variations in liver iron metabolism and deposition have been reported for different mouse inbred lines [38]. We assume that deposition of iron in the liver differing between mouse strains and possibly rodent species might be one of the reasons for the inconsistencies within MRR investigations of experimental liver fibrosis and cirrhosis published to date.

## 5. Conclusion

In toxic fibrosis, iron deposition in the liver is directly linked to CCl_4_ induced damage.

Differences in T1, T2^*∗*^, and T1/T2^*∗*^ between different aetiologies of liver fibrosis might be useful for discriminating distinct pathophysiologies by MRR.

Individual genetic variations of iron metabolism in humans might also influence MRR determination of disease severity in patients with liver disease.

## Figures and Tables

**Table 1:** Histologic criteria for centro-central lobular fibrosis in CCl_4_-induced animal models.

Histologic findings	*F*-score
No fibrosis	0
Perivenular fibrosis (+ onset of bridging)	1
Venocircumferential fibrosis (+ incomplete bridging)	2
Centro-central complete bridging	3
Distinct and broad complete bridging	4

**Table 2:** Histologic criteria for fibrosis staging in bile duct ligation and comparable animal models.

Histologic findings	*F*-score
No fibrosis	0
Scattered periportal and perineoductular fibrosis (incomplete lamellae)	1
Periportal, perineoductular fibrosis (complete lamellae) +/− beginning septa	2
Periportal, perineoductular fibrosis with portal-portal septa	3
Complete cirrhosis	4
